# *Camchaya khongchiamensis* Chatan & Promprom (Asteraceae: Vernonieae), a New Species From Thailand

**DOI:** 10.1002/ece3.71197

**Published:** 2025-03-27

**Authors:** Wilawan Promprom, Phukphon Munglue, Wannachai Chatan

**Affiliations:** ^1^ Department of Biology Faculty of Science, Mahasarakham University Kantharawichai Thailand; ^2^ Plant and Innovation Research Unit Mahasarakham University Kantharawichai Thailand; ^3^ Program of Biology Faculty of Science, Ubon Ratchathani Rajabhat University Ubon Ratchathani Thailand

**Keywords:** classification, conservation assessment, diversity, eastern Thailand, endangered species, flowering plant, plant diversity, taxonomy, Ubon Ratchathani

## Abstract

*Camchaya khongchiamensis* Chatan & Promprom (Asteraceae), a new species from Ubon Ratchathani Province, Thailand, is described and illustrated. It is similar to *C. loloana*, but clearly different from *C. loloana* by having a larger size of capitula, involucre, receptacle, and corolla lobe. In addition, it mostly has fewer florets (18–40 [−50]) per capitulum; lower haft whitish, upper haft purplish corolla tubes; sagittate base anthers; and white except for the purple near apex style. A distribution map, pollen morphology, and a table with comparative morphological characters of the new species and its closest relative are provided, as well as the preliminary conservation assessment under the IUCN criteria.

## Graphical abstract

Camchaya khongchiamensis Chatan & Promprom (Asteraceae), a new species from Ubon Ratchathani Province, Thailand. A distribution map, pollen morphology, and a table with comparative morphological characters of the new species and its closest relative are provided, as well as the preliminary conservation assessment under the IUCN criteria.

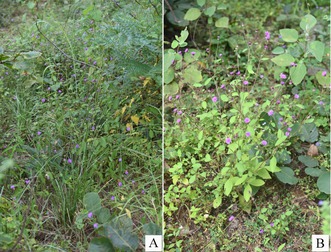

## 1 Introduction

The genus *Camchaya* Gagnep. (Asteraceae) was established in 1920 (Gagnepain 1920) and currently consists of 10 species (Noyori et al. 2022; POWO 2024). It is a member of the subtribe Centrapalinae, which belongs to the tribe Vernonieae (Susanna et al. 2020). Its characteristics are defined by its leafy stems, involucres with phyllaries that are not reflexed, a lack of a carpopodium in cypsela, a pappus arranged in a single series (which can be deciduous or sometimes absent), and pollen with six apertures.

As *Camchaya* is a member of the Asteraceae family, which is known for its use in treating various ailments such as inflammation, ulcers, and gastrointestinal disorders, it is also rich in bioactive compounds such as flavonoids, phenolic acids, and terpenoids. These compounds contribute to its medicinal properties. Additionally, specific phytochemicals like inulin, known for its prebiotic effects, are commonly found in this family, enhancing their nutritional and therapeutic potential (Sharma et al. 2022). However, studies specifically focusing on *Camchaya* remain limited, and further scientific research is needed. The genus is closely related to *Koyamasia* Robinson (Robinson 1999), but can be differentiated by its not reflexed phyllaries, obovate cypselae, and hexaporate pollen, as opposed to *Koyamasia*'s reflexed phyllaries, oblong cypselae and three‐apertured pollen (Bunwong et al. 2014). *Camchaya* species are primarily found in Southeast Asia, extending into China, and often grow on forest floors or rocky areas in Indochina's dipterocarp forests (Bunwong et al. 2014). In Thailand, there are seven species including a new species, *Camchaya thailandica* Bunwong, Chantar. & S.C.Keeley, which was reported in 2012 (Bunwong et al. 2012, 2014; Koyama et al. 2016).

In the past 10 years, the authors have conducted taxonomic work in Eastern and Northeastern Thailand and discovered many new plant taxa in these regions (Chatan and Promprom 2017, 2018a, 2018b; Promprom and Chatan 2018, 2020, 2021, 2022; Promprom et al. 2023, 2024a, 2024b). However, the authors have never conducted research in Khongchiam district in Ubon Ratchathani Province, Thailand. Khongchiam is located near the border of Thailand and Laos (Lao PDR). It is a relatively unexplored region in eastern Thailand, known for its remarkable diversity of plant species, particularly those in the Asteraceae family. Khong Chiam District boasts a distinctive ecological landscape, comprising both dry evergreen and deciduous dipterocarp forests. The biodiversity of this area is further enhanced by the presence of two significant rivers, the Mekong and the Moon, which flow through the district. During the field works conducted in Khongchiam district from 2023 to 2024, the authors found a previously unidentified species within the Asteraceae. Using herbarium specimens and taxonomic studies, the investigation of key morphological characters—such as the capitula, receptacle, corolla, anther, and style—revealed unique traits that set this species apart from previously known *Camchaya* species. As a result, the species was formally described and named *C. khongchiamensis* Chatan & Promprom. Along with its scientific description, a vernacular name, ecological details, and a preliminary conservation assessment were also provided.

## 2 Materials and Methods

Digital images of herbarium specimens from BK, BKF, K, L, P, and US herbaria on JSTOR Global Plants (https://plants.jstor.org/) and the herbaria's websites were obtained and analyzed. A critical examination of these plants was conducted through comparisons with relevant taxonomic literature, including Gagnepain (1920), Kerr (1935), Koyama (1984), Robinson (1999), and Bunwong et al. (2009, 2012, 2014), as well as Koyama et al. (2016). The morphological description is based on both herbarium specimens and field observations, with terminology adhering to these sources. Additionally, the morphological terminology follows Harris and Harris (1994). Floral dissections and measurements were performed under a stereo microscope using a micrometer or ruler, and measurements were taken from herbarium specimens collected by the authors during fieldwork. The new species shows morphological similarities to C. *loloana* Dunn ex Kerr, including var. *loloana*, var. *mukdahanensis* H. Koyama, and var. *pseudotenuiflora* H. Koyama (POWO 2024). Consequently, type specimens and protologues of these taxa (Kerr 1935; Koyama 1984) were consulted during the investigation.

The pollen of *C. khongchiamensis* Chatan & Promprom sp. nov. was studied and examined by using the method from Erdtman (1972). The pollen samples were dehydrated through a series of ethanol solutions at concentrations of 70%, 80%, 95%, and 100%, with each step taking 5 min. After that, they were left to air‐dry overnight at room temperature. The dried pollen was then mounted onto aluminum panels, which were attached to stubs using carbon tape. We studied the pollen grains with a scanning electron microscope (SEM) (Hitachi, TM‐4000plus, Hitachi High‐Tech, Tokyo, Japan) at the Laboratory Equipment Center in Mahasarakham University's Division of Research Facilitation and Dissemination and took photomicrographs for further analysis.

The conservation status was assessed following the Guidelines for Using the IUCN Red List Categories and Criteria, Version 16 (IUCN Standards and Petitions Committee 2024). This evaluation aligns with global standards and provides a reliable framework for classifying the species' conservation requirements.

## 3 Taxonomic Treatment

### 3.1 *Camchaya khongchiamensis* Chatan & Promprom Sp. Nov

Figures 1, 2, 3, 4.

**FIGURE 1:**
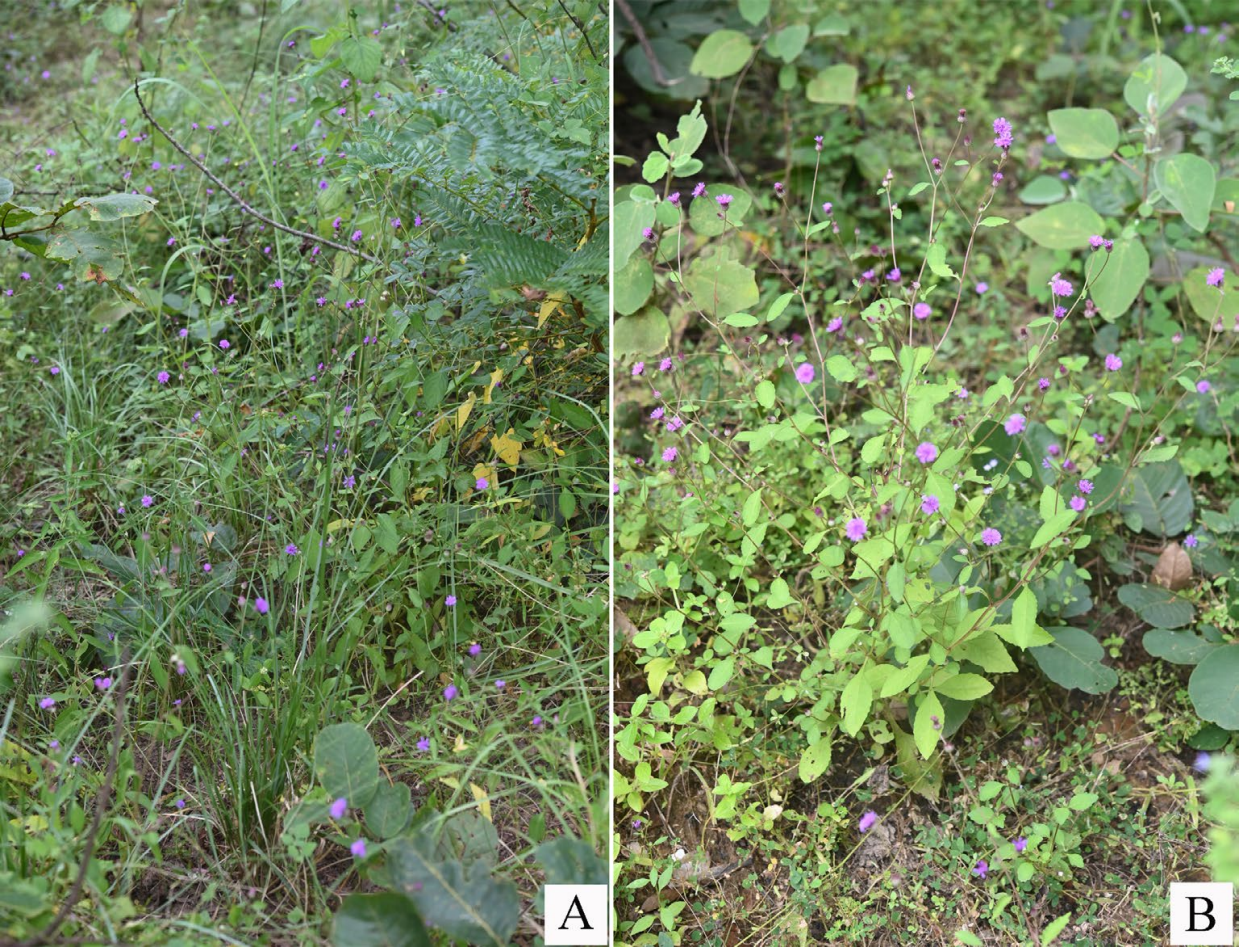
*Camchaya khongchiamensis* Chatan & Promprom: (A) Habitat; (B) Habit.

**FIGURE 2:**
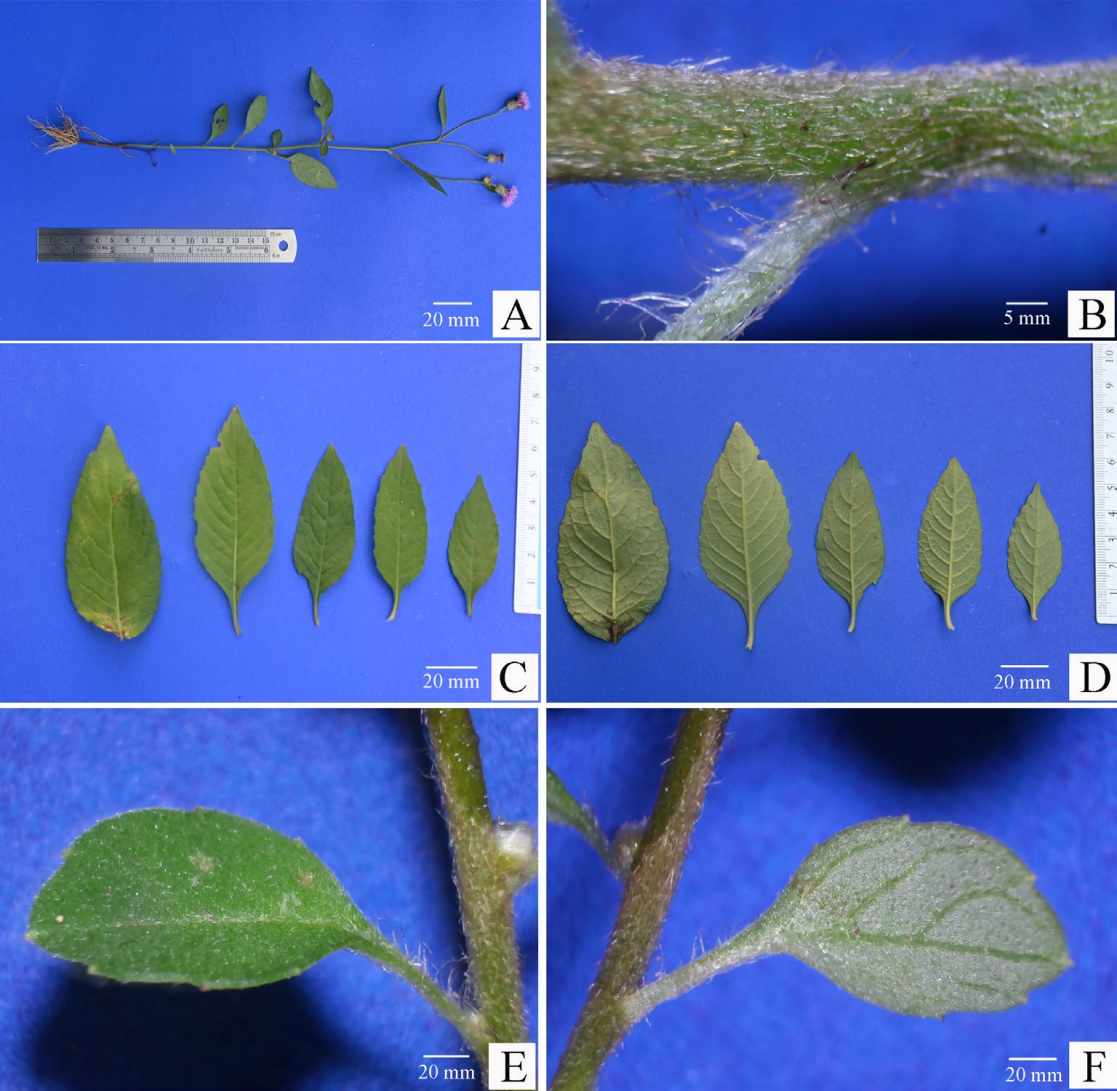
*Camchaya khongchiamensis* Chatan & Promprom. (A) Whole plant; (B) Stem and parts of petiole; (C) Leaves (upper leaf surface); (D) Leaves (under side); (E, F) Upper and lower leaf surfaces, respectively.

**FIGURE 3:**
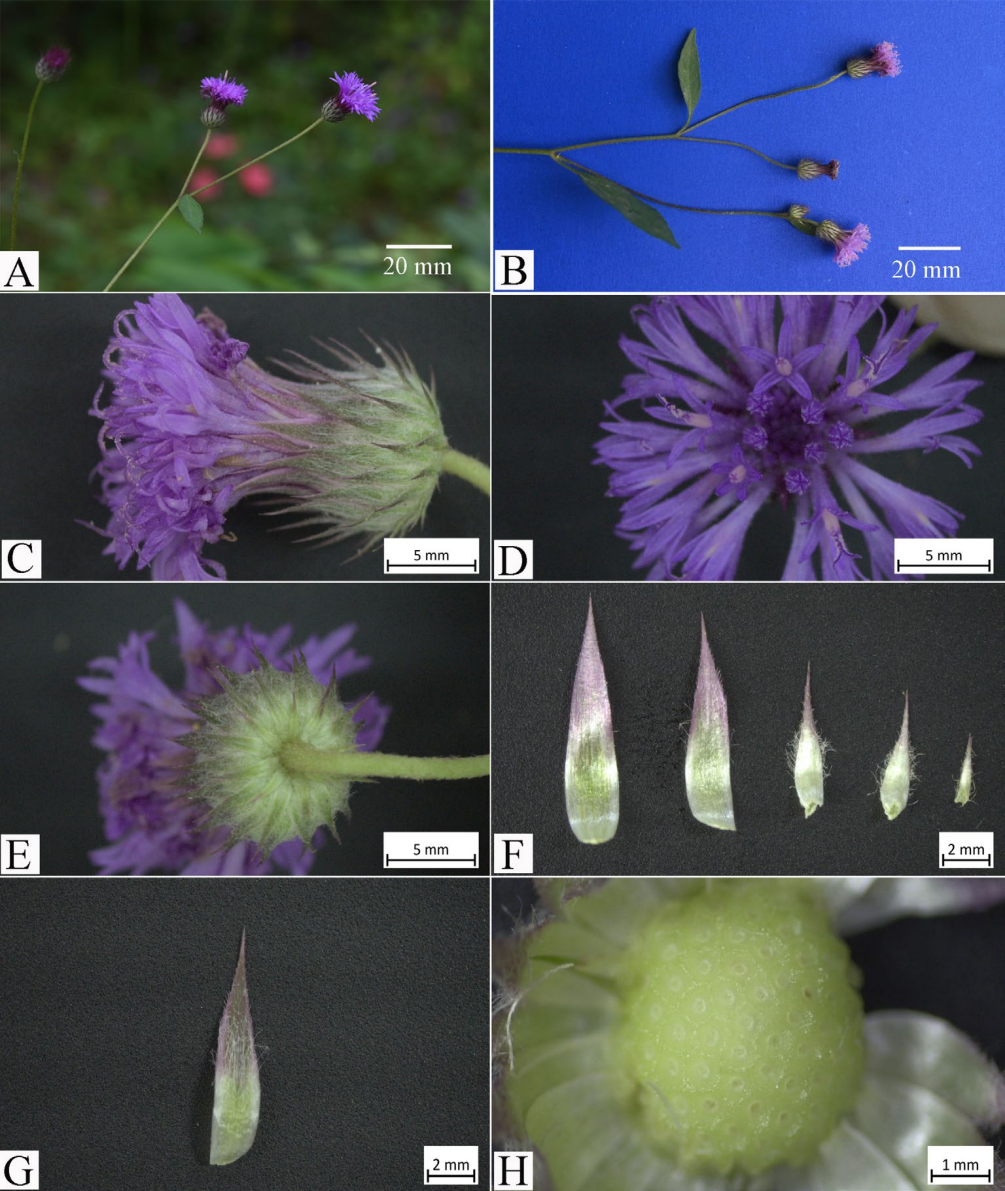
*Camchaya khongchiamensis* Chatan & Promprom. (A, B) Capitulescences and capitula; (C–E) capitula in lateral, top, and bottom views, respectively; (F) upper side of phyllaries (from left to right: Inner ones to outer ones); (G) under side of phyllary (inner one); (H) receptacle.

**FIGURE 4:**
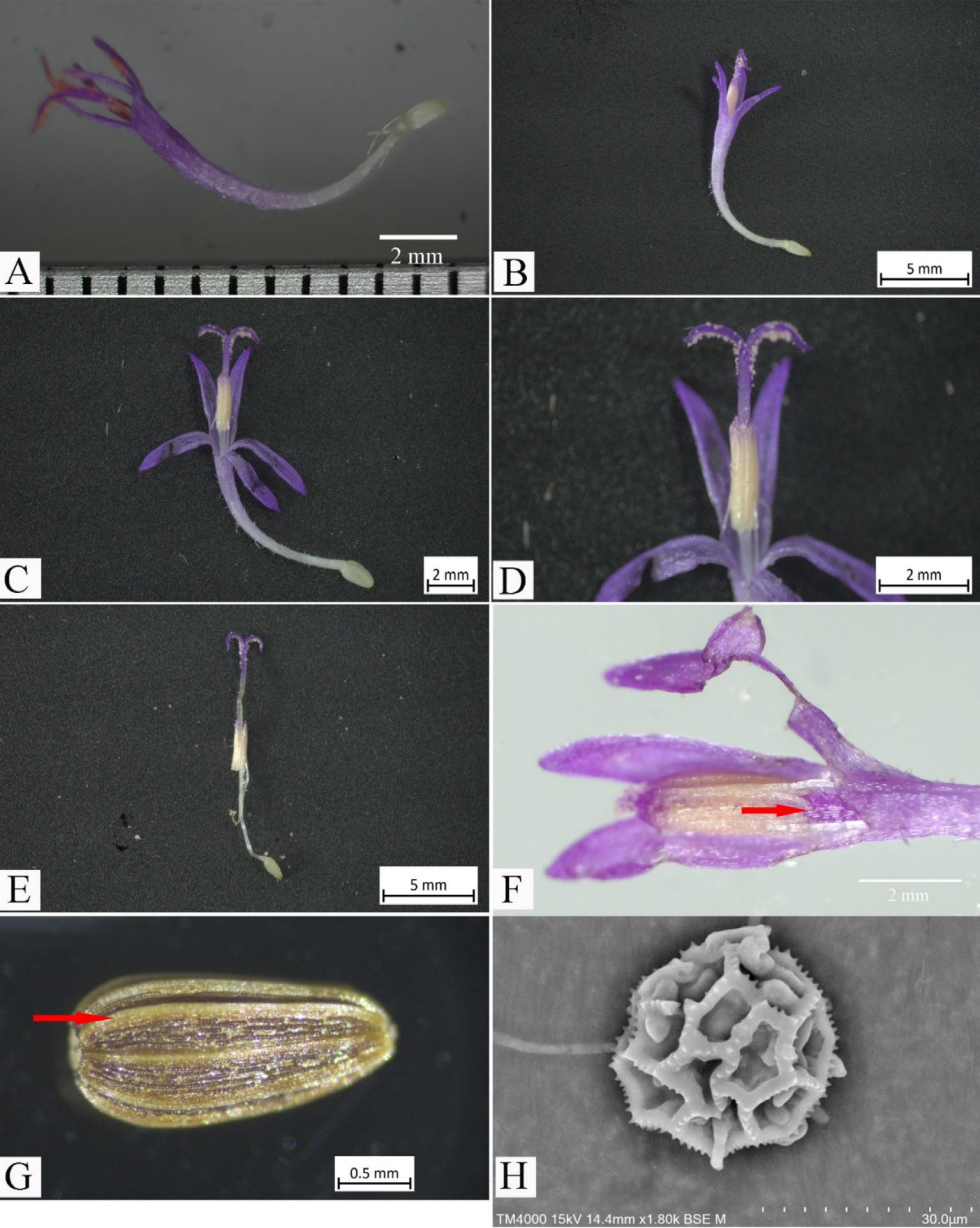
*Camchaya khongchiamensis* Chatan & Promprom. (A, B) Florets; (C, D) florets (the open top of the corolla showing the upper parts of the stamen and pistil); (E) floret (corolla removed); (F) anthers (the sagittate base indicated by arrowhead); (G) cypsela (rib indicated by arrowhead); (H) pollen.

**FIGURE 5:**
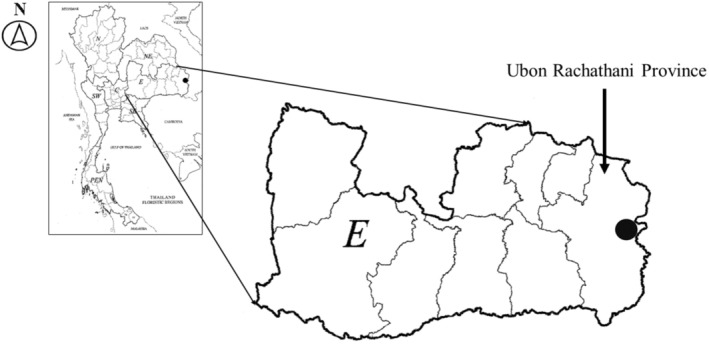
Distribution of *Camchaya khongchiamensis* Chatan & Promprom (black circle) in Khong Chiam District, Ubon Ratchathani Province, Thailand.

### 3.2 Type

Thailand, Khong Chiam District, Ubon Ratchathani Province, alt. 105–110 m, 15°18′39.1″ N 105°30′09.2″ E, November 10, 2023, W.Chatan 2888 (holotype: K!; isotype: BK!).

### 3.3 Diagnosis

*Camchaya khongchiamensis* is morphologically similar to *C. loloana* in the presence of glandular hairs and ≤ 1 mm long marginal spines on phyllaries and 10‐ribbed cypselae, but differs from the latter by its larger size of capitula (15–21 mm vs. 9–10 mm long in anthesis), involucres (12–14 mm long vs. 7–10 mm long), receptacles (4.2–4.5 mm vs. 2–4 mm diam.), corolla lobes (4–5 vs. 1.5–3 mm). In addition, *C. khongchiamensis* has 18–40 (−50) florets per capitulum; lower haft whitish, upper haft purplish corolla tubes; sagittate base anthers; and white except for the purple near apex style, while *C. loloana* has 30–100 florets per capitulum; purple or rarely white corolla tubes; rounded base anthers; and the purple styles except for a short white part of thebase.

### 3.4 Description

Annual herbs, up to 70 cm tall. Stem erect in the middle and upper part, terete, inconspicuously ribbed, 1–5 mm in diam.; surface densely covered with T‐shape hairs, glandular hairs, and filiform hairs. Leaves alternate; petiole 0.4–1 cm long; blades ovate, occasionally lanceolate, 3–8 × 2–4 cm, apex acute or obtuse, base attenuate, margin subentire or serrate with approximately 1–2 mm long teeth on each side, chartaceous, green adaxially, pale greenish gray abaxially; both surfaces sparse short filiform hairs, whip‐shaped hairs, and glands, few with T‐shape hairs present; midrib, lateral veins, and other veins slightly prominent on the adaxial side and more visible on the abaxial side. Capitulescences terminal and axillary, solitary or in panicles. Capitula 15–21 mm long in anthesis. Peduncles up to 20 cm long. Involucres campanulate, 12–14 × 10–12 mm. Phyllaries 7–10 seriate, imbricate; each linear or oblong, 0.27–2.3 × 3–10.5 mm, apex upward to a capitular apex, dull gray‐green with pale purple apices, margins with spines to 0.1–0.3 mm long, glabrous adaxially, hairy with T‐shape and glandular abaxially, margins with up to 0.5 mm long spines, apex aristate, all phyllaries lanceolate, 3.0–10.5 × 0.5–2.2 mm; Receptacle convex, 4–4.5 mm diam., glabrous. Florets 18–40 (−50) per capitulum; corollas tubular‐infundibular, sparsely glandular hairy; tubes 6.8–8 mm long, lower haft whitish, upper haft purplish; lobes 5, narrowly oblong, 4–5 × 0.6–0.8 mm, apex acute. Stamens 5, anthers lanceolate, 1.8–2 mm long, apex acute, base sagittate, apical appendage triangular, ca. 0.3–0.4 mm long, apex acute; filaments ca. 5–6 mm long, white, glabrous, connate to the corolla near the corolla mouth, free part 1–1.2 mm long. Ovary obovoid, 0.8–1.3 × 0.5–0.8 mm; styles linear, 8–9 mm long, white except for the purple near the apex, sparsely white hairs near the apex; stigmas 2‐branched, branches 1–1.3 mm long, sparsely purplish hairs. Cypselae obovoid, 1.6–2.4 mm long, ca. 1 mm diam., 10‐ribbed, without carpopodium. Pappus bristles, 1.5–1.8 mm long, white, thin and filiform, caducous, with minute white spine, or absent. Pollen spheroidal, echinolophate, 6‐porate, without micropuncta, 25.5–32.7 μm diam.

### 3.5 Phenology

Flowering October to November, fruiting November to December.

### 3.6 Etymology

The specific epithet “khongchiamensis” refers to the type locality, Khong Chiam District.

### 3.7 Distribution

Thailand (Khong Chiam District, Ubon Ratchathani Province, the Eastern Thailand) (Figure 5).

### 3.8 Habitat and Ecology

The new species grows on both dry and slightly wet soil over exposed areas in dry‐dipterocarp forest.

### 3.9 Vernacular Name

Phu Muang Ngam.

### 3.10 Preliminary Conservation Status

The new species has so far been identified only in its type locality. The plant is estimated to number fewer than 2500 mature individuals and mature individuals in each subpopulation fewer than 250. Therefore, it should be considered as “Endangers (EN)” according to IUCN criteria C(C2ai) (IUCN Standards and Petitions Committee 2024).

### 3.11 Additional Specimens Examined (Paratypes)

Thailand: Ubon Ratchathani Province, Khong Chiam District: 15°18′39.1″ N 105°30′09.3″ E, November 11, 2023 (*W.Chatan 2889*: BK); 15°18′39.1″ N 105°30′09.3″ E, November 11, 2023 (*W.Chatan 3890*: BK); 15°18′40.6″ N 105°30′03.8″ E, November 11, 2024 (*W.Chatan 3891*: BK); 15°18′29.8″ N 105°29′38.7″ E, October 5, 2024 (*W.Chatan 3892*: BK); 15°18′25.0″ N 105°29′35.8″ E, October 5, 2024 (*W.Chatan 3893*: BK); 15°18′25.5″ N 105°29′37.1″ E, October 5, 2024 (*W.Chatan 3894*: BK); 15°18′29.1″ N 105°29′44.8″ E, October 6, 2024 (*W.Chatan 3895*: BK); 15°17′31.7″ N 105°29′21.9″ E, October 6, 2024 (*W.Chatan 3896*: BK).

### 3.12 Notes

*Camchaya khongchiamensis* Chatan & Promprom is different from *C. loloana* (including three varieties, i.e., var. *loloana*, var. *mukdahanensis* H.Koyama, and var. *pseudotenuiflora* H.Koyama), another species of the genus distributed in Cambodia, China South‐Central, Laos, Thailand, and Vietnam (Koyama 1984; POWO 2024). The details of the morphological differences between the two plant species are significant and can be categorized into various characteristics (Table 1).

**TABLE 1:** Morphological differences between *Camchaya khongchiamensis* Chatan & Promprom and *C. loloana* Dunn *ex* Kerr.

Characters	*Camchaya khongchiamensis*	*Camchaya loloana*
1. Blade leaf apex	Acute or obtuse	Acute or acuminate
2. Blade margin	Serrate or subentire	Serrate or undulate
3. Capitulescences	Solitary or grouped in panicle	Grouped in panicle
4. Capitula	15–21 mm long in anthesis	9–10 mm long in anthesis
5. Involucres	12–14 mm long	7–10 mm long
6. Phyllaries	7–10 seriate, lanceolate or linear‐oblong; apex upward to a capitular apex; marginalspines 0.1–0.3 mm long	5–8‐seriate, lanceolate or oblong or linear‐oblong; apex downward or slightly spread out; marginalspines up to 1 mm long
7. Receptacles	4.2–4.5 mm diam.	2–4 mm diam.
8. Florets	18–40 (−50) per capitulum	30–100 per capitulum
9. Corollas tubes	6.8–8 mm long, lower haft whitish, upper haft purplish	Ca. 5.5–9.5 mm long, purple or rarely white
10. Corolla lobes	4–5 mm long	1.5–3 mm long
11. Anther base	Sagittate	Rounded
12. Styles	White, except for the purple near the apex and branches	Whole part purple, except for a short white section at the base.
13. Cypselae	1.6–2.4 mm long	1.3–5 mm long
14. Pappus	1.5–1.8 mm long	1.5–3 mm long

*Camchaya khongchiamensis* has capitula that are either solitary or part of a paniculate structure (up to 10 capitulae), measuring 15–21 mm in length during anthesis. In contrast, *C. loloana* consistently exhibits paniculate capitulescences, with capitula measuring 9–10 mm in length during anthesis. However, the number of capitula in a panicle can reach up to 10, as observed in *C. khongchiamensis*.

The involucres of the new species are longer (12–14 mm) in length, whereas those of *C. loloana* range from 7 to 10 mm. Phyllaries in the new species are arranged in 7–10 series and feature shorter marginal spiny elements measuring 0.1–0.3 mm. In contrast, *C. loloana* has phyllaries arranged in 5–8 series with spiny elements up to 1 mm long. The receptacle diameter of the new species is generally larger (4.2–4.5 mm) compared to 2–4 mm in *C. loloana*. The new species has 18–40 (rarely up to 50) florets per capitulum, while *C. loloana* has 30–100 florets per capitulum.

The corolla tubes of the new species measure 6.8–8.0 mm in length, with a whitish lower portion and a purplish upper portion. In contrast, *C. loloana* has corolla tubes approximately 5.5–9.5 mm long, which are predominantly purple but may occasionally be white. The corolla lobes in the new species are 4–5 mm long compared to 1.5–3 mm in *C. loloana*. Anthers in the new species are 1.8–2 mm long with a sagittate base, while those in *C. loloana* range from 1.5 to 2.5 mm long with a rounded base. Styles in the new species are 8–9 mm long and predominantly white except for a purple near the apex. In *C. loloana*, styles range from 5 to 11 mm and are purple. The cypselae of the new species measure 1.6–2.4 mm, whereas those of *C. loloana* are 1.3–5 mm long. The pappus of the new species has pappus lengths of 1.5–1.8 mm and *C. loloana* ranges from 1.5 to 3 mm.

These detailed morphological differences highlight the distinct characteristics that differentiate *C. khongchiamensis* from *C. loloana*, contributing to a clearer understanding of these distinct species. The key to the species of *Camchaya* in Thailand, modified from Koyama et al. (2016), is presented as follows.

### 3.13 Key to the Species of *Camchaya* in Thailand

**Table:** 

1	Phyllary margin without spines	2
—	Phyllary margin spinose	3
2	Cypselae 4–5‐ribbed	*C. gracilis*
—	Cypselae 10‐ribbed	*C. thailandica*
3	Cypselae 5 − (6–9)‐ribbed	*C. pentagona*
—	Cypselae 10‐ribbed	4
4	Phyllaries eglandular, marginal spines to 10 mm	*C. spinulifera*
—	Phyllaries with glands, marginal spines to 5 mm	5
5	Phyllaries acuminate; cypselae 2.5–3 mm long	*C. kampotensis*
5	Phyllaries aristate or apiculate; achenes 1.5–2 mm long	6
6	Leaves without T‐shaped hairs; phyllaries spinose ≥ 1 mm long	*C. tenuiflora*
—	Leaves with T‐shaped hairs; phyllaries spinose ≤ 1 mm long	7
7	Corolla lobes 1.5–3 mm long, anther base rounded	*C. loloana*
—	Corolla lobes 4–5 mm long, anther base sagittate	*C. khongchiamensis*

## Author Contributions

**Wilawan Promprom:** data curation (equal), formal analysis (equal), funding acquisition (equal), investigation (equal), methodology (equal), project administration (equal), resources (equal), validation (equal), writing – original draft (equal), writing – review and editing (equal). **Phukphon Munglue:** data curation (equal), formal analysis (equal), investigation (equal), methodology (equal), resources (equal), validation (equal), visualization (equal), writing – original draft (equal). **Wannachai Chatan:** conceptualization (lead), data curation (equal), formal analysis (equal), funding acquisition (equal), investigation (equal), methodology (equal), project administration (equal), resources (equal), supervision (equal), visualization (equal), writing – original draft (equal), writing – review and editing (equal).

## Conflicts of Interest

The authors declare no conflicts of interest.

## Data Availability

The authors have nothing to report.

## References

[ece371197-bib-0001] Bunwong, S. , P. Chantaranothai , and S. C. Keeley . 2012. “A New Species of Camchaya (Asteraceae, Vernonieae) From Thailand.” PhytoKeys 12: 53–57. 10.3897/phytokeys.12.3221. PMC3349056 22645414

[ece371197-bib-0002] Bunwong, S. , P. Chantaranothai , and S. C. Keeley . 2014. “Revisions and Key to the Vernonieae (Compositae) of Thailand.” PhytoKeys 37: 25–101. 10.3897/phytokeys.37.6499. PMC4023333 24843297

[ece371197-bib-0003] Bunwong, S. , H. Robinson , and P. Chantaranothai . 2009. “Taxonomic Notes on *Camchaya* and *iodocephalus* (Vernonieae: Asteraceae), and a New Genus *iodocephalopsis* .” Proceedings of theBiological Society of Washington 122, no. 3: 357–363. 10.2988/08-45.1.

[ece371197-bib-0004] Chatan, W. , and W. Promprom . 2017. “New Subspecies of *Ardisia crenata* (Primulaceae) From Thailand.” Taiwania 62, no. 2: 116–120. 10.6165/tai.2017.62.116.

[ece371197-bib-0005] Chatan, W. , and W. Promprom . 2018a. “New Medicinal Plant Variety of *Trichosanthes tricuspidata* Lour. (Cucurbitaceae) From Northeastern Thailand.” Pharmacognosy Journal 10, no. 1: 29–32. 10.5530/pj.2018.1.6.

[ece371197-bib-0006] Chatan, W. , and W. Promprom . 2018b. “New Species of *Bauhinia* (Cercidoideae: Leguminosae) From Thailand.” Phytotaxa 385, no. 1: 43–47. 10.11646/phytotaxa.385.1.6.

[ece371197-bib-0007] Erdtman, G. 1972. Pollen Morphology and Plant Taxonomy: Angiosperms (An Introduction to Palynology. I); Corrected Reprint of the Edition of 1952 With a New Addendum, 1–539. Hafner Publication Company.

[ece371197-bib-0008] Gagnepain, F. 1920. “ *Camchaya* .” Notulae Systematicae 4: 14–15.

[ece371197-bib-0009] Harris, J. G. , and M. W. Harris . 1994. Plant Identification Terminology: An Illustrated Glossary, 1–206. Spring Lake Publishing.

[ece371197-bib-0010] IUCN Standards and Petitions Committee . 2024. “Guidelines for Using the IUCN Red List Categories and Criteria.” Version 16. Prepared by the Standards and Petitions Committee. https://www.iucnredlist.org/documents/RedListGuidelines.pdf.

[ece371197-bib-0011] Kerr, A. H. G. 1935. “ *Camchaya loloana* .” Bulletin of Miscellaneous Information (Royal Botanic Gardens, Kew) 1935: 327.

[ece371197-bib-0012] Koyama, H. 1984. “Taxonomic Studies in the Compositae of Thailand 3.” Acta Phytotaxo‐Nomica et Geobotanica 35: 49–58. 10.18942/bunruichiri.KJ00001078487.

[ece371197-bib-0013] Koyama, H. , S. Bunwong , P. Pornpongrungrueng , and H. D. J. Nicholas . 2016. “Compositae (Asteraceae).” In Flora of Thailand, edited by T. Santisuk and H. Balslev , vol. 13, 143–428. Forest Herbarium, Royal Forest Department.

[ece371197-bib-0014] Noyori, W. , N. Komada , P. Souladeth , and S. Tagane . 2022. “Camchaya Bolavenensis (Asteraceae: Vernonieae), a New Species From Bolaven Plateau, Southern Laos.” Phytotaxa 536, no. 1: 1–6. 10.11646/phytotaxa.536.1.1.

[ece371197-bib-0015] POWO . 2024. “Plants of the World Online. Facilitated by the Royal Botanic Gardens, Kew.” http://www.plantsoftheworldonline.org/.

[ece371197-bib-0016] Promprom, W. , and W. Chatan . 2018. “ *Sindora stipitata* (Detarioideae, Leguminosae), a New Species From Thailand.” PhytoKeys 100: 23–30. 10.3897/phytokeys.100.25870. PMC6023954 29962894

[ece371197-bib-0017] Promprom, W. , and W. Chatan . 2020. “A New Species of *Dischidia* (Apocynaceae, Asclepiadoideae) From North‐Eastern Thailand.” PhytoKeys 144: 23–30. 32231459 10.3897/phytokeys.144.47977 PMC7093575

[ece371197-bib-0018] Promprom, W. , and W. Chatan . 2021. “ *Stemona namkhunensis* (Stemonaceae), a New Species From Eastern Thailand.” Taiwania 66, no. 3: 332–336. 10.6165/tai.2021.66.332.

[ece371197-bib-0019] Promprom, W. , and W. Chatan . 2022. “Acilepis Nakhonphanomensis (Asteraceae), a New Species From Northeastern Thailand.” Taiwania 67, no. 4: 571–575. 10.6165/tai.2022.67.571.

[ece371197-bib-0020] Promprom, W. , W. Chatan , P. Pasorn , N. Prasertsri , and T. Angkahad . 2023. “A New Species of *Amorphophallus* (Araceae) From Northeastern Thailand.” PhytoKeys 229: 131–138. 10.3897/phytokeys.229.106466. 37485010 PMC10359917

[ece371197-bib-0021] Promprom, W. , P. Munglue , and W. Chatan . 2024a. “ *Alocasia sakonakhonensis* (Araceae), a New Species From Northeastern Thailand.” Ecology and Evolution 14, no. 5: e11462. 10.1002/ece3.11462. 38799389 PMC11117049

[ece371197-bib-0022] Promprom, W. , P. Munglue , and W. Chatan . 2024b. “ *Ardisia crenata* Subsp. Mukdahanensis, a New Subspecies of Primulaceae From Thailand.” PhytoKeys 247: 1–10. 10.3897/phytokeys.247.126743. 39372658 PMC11450457

[ece371197-bib-0023] Robinson, H. E. 1999. “Revisions in Paleotropical Vernonieae (Asteraceae).” Proceedings of Biological Society of Washington 112: 220–247.

[ece371197-bib-0024] Sharma, M. , N. Bithel , and M. Sharma . 2022. “Ethnobotany, Phytochemistry, Pharmacology and Nutritional Potential of Medicinal Plants From Asteraceae Family.” Journal of Mountain Research 17, no. 2: 67–82. 10.51220/jmr.v17i2.7.

[ece371197-bib-0025] Susanna, A. , B. G. Baldwin , R. J. Bayer , et al. 2020. “The Classification of the Compositae: A Tribute to Vicki Ann Funk (1947–2019).” Taxon 69, no. 4: 807–814. 10.1002/tax.12235.

